# Oxidative and Nitrosative Stress Biomarkers in Personnel of Radiodiagnostic Laboratory Services Exposed to Ionizing Radiation

**DOI:** 10.7759/cureus.103923

**Published:** 2026-02-19

**Authors:** Unal Öztürk, Mujde Aksimsek, Ergul Belge Kurutas

**Affiliations:** 1 Cardiology, Kahramanmaras Sutcu Imam University, Kahramanmaras, TUR; 2 Bioengineering, Kahramanmaras Sutcu Imam University, Kahramanmaras, TUR; 3 Biochemistry, Kahramanmaras Sutcu Imam University, Kahramanmaras, TUR

**Keywords:** biomarkers, ionizing radiation, nitrosative stress, oxidative stress, radiology personnel

## Abstract

Background: Ionizing radiation (IR) is widely used in medical imaging and interventional procedures. However, chronic occupational exposure may result in oxidative and nitrosative stress, contributing to long-term health risks.

Objective: This study aimed to evaluate oxidative and nitrosative stress biomarkers in radiodiagnostic laboratory personnel occupationally exposed to IR compared to non-exposed healthy controls.

Methods: A cross-sectional study was conducted, including 100 radiodiagnostic laboratory personnel (exposed group) and 100 age- and sex-matched healthy controls. Venous blood samples were analyzed for catalase (CAT), superoxide dismutase (SOD), malondialdehyde (MDA), nitric oxide (NO), and 3-nitrotyrosine (3-NTx). Standard spectrophotometric and enzyme-linked immunosorbent assay (ELISA) methods were used. Group comparisons were performed using Student’s t-test and chi-square test, with p<0.05 considered statistically significant.

Results: The exposed group demonstrated significantly lower CAT activity (18.25 ± 4.07 vs. 22.46 ± 4.34 U/mg Hb, p=0.001), higher SOD activity (210.52 ± 32.38 vs. 180.21 ± 29.71 U/mg Hb, p=0.004), and elevated MDA levels (4.84 ± 1.21 vs. 3.12 ± 0.92 µmol/L, p=0.002). Moreover, NO (35.74 ± 6.81 vs. 26.05 ± 5.67 µmol/L, p=0.003) and 3-NTx (18.41 ± 3.59 vs. 12.89 ± 3.14 ng/mL, p=0.001) levels were significantly higher in the exposed group compared to controls.

Conclusion: Occupational exposure to ionizing radiation is associated with increased oxidative and nitrosative stress, reflected by altered biomarker profiles in radiodiagnostic laboratory personnel. These findings highlight the importance of strict radiation protection measures and suggest a potential role for biomarker monitoring and antioxidant interventions to safeguard worker health.

## Introduction

Ionizing radiation (IR) has become an indispensable tool in modern medicine, particularly in diagnostic and interventional radiology. With the increasing use of radiological modalities such as X-ray, computed tomography (CT), and angiographic imaging, healthcare professionals working in radiodiagnostic laboratories are continuously exposed to low doses of radiation. Although these exposures are typically within recommended safety limits, cumulative effects over time may lead to significant biological consequences [1,2].

The primary mechanism of IR-induced biological damage involves the generation of reactive oxygen species (ROS) and reactive nitrogen species (RNS) through radiolysis of water and interaction with biomolecules. Excessive production of these free radicals overwhelms the antioxidant defense systems of the cell, resulting in oxidative and nitrosative stress. This imbalance may lead to structural and functional alterations in lipids, proteins, and DNA, thereby contributing to mutagenesis, apoptosis, and carcinogenesis [3,4].

Oxidative stress is commonly assessed by measuring lipid peroxidation products such as malondialdehyde (MDA) and enzymatic antioxidants, including catalase (CAT) and superoxide dismutase (SOD). Nitrosative stress, on the other hand, can be evaluated by nitric oxide (NO) and 3-nitrotyrosine (3-NTx) levels, which reflect RNS-mediated protein modifications [5]. Several studies have suggested that chronic low-dose radiation exposure may disrupt oxidant/antioxidant homeostasis in healthcare workers, but data remain limited, particularly in regions with varying radiation safety practices [6,7].

The Eastern Mediterranean region, encompassing provinces such as Adana, Hatay, and Kahramanmaraş in Türkiye, hosts a considerable number of radiodiagnostic laboratories where healthcare workers are routinely exposed to IR. Despite the widespread use of radiological equipment in these centers, there is a paucity of data on occupational oxidative and nitrosative stress biomarkers in this population.

Therefore, the present study aimed to determine the levels of oxidative and nitrosative stress biomarkers in radiodiagnostic laboratory personnel in the Eastern Mediterranean region of Türkiye. By comparing radiation-exposed healthcare workers with unexposed controls, this study sought to provide evidence on the potential health risks associated with occupational IR exposure and to emphasize the importance of radiation protection practices.

## Materials and methods

Study design and population

This cross-sectional study was conducted between August 2020 and June 2021 in the Eastern Mediterranean region of Türkiye, covering the provinces of Adana, Hatay, and Kahramanmaraş (Appendices A-C). A total of 200 participants were recruited, including 100 radiodiagnostic laboratory personnel occupationally exposed to ionizing radiation (IR) and 100 age- and sex-matched healthy controls with no known radiation exposure. The exposed group consisted of radiology technicians, interventional radiology staff, and angiography laboratory personnel.

The sample size was calculated using G*Power software (Ver. 3.1, Heinrich-Heine-Universität Düsseldorf, Düsseldorf, Germany) based on an effect size of 0.5, a power of 90%, and α=0.05. This estimation was supported by previous studies investigating oxidative stress biomarkers in radiation-exposed healthcare workers [8,9]. Inclusion criteria for the exposed group were: (i) active employment in radiodiagnostic laboratories for at least one year, and (ii) willingness to participate with informed consent. Exclusion criteria included: (i) history of chronic systemic disease (e.g., diabetes mellitus, chronic kidney disease, or autoimmune disorders), (ii) use of antioxidant supplements or medications within the last three months, and (iii) prior history of therapeutic radiation exposure. The same exclusion criteria were applied to the control group. All participants completed a structured questionnaire (Appendix D) prepared by the investigators, including sociodemographic information (age, sex, education, income, marital status), lifestyle habits (smoking, alcohol use), occupational history (work duration, radiation exposure site), and protective measures (use of lead apron, thyroid shield, and dosimeter). Health-related complaints such as fatigue, headache, irritability, and reproductive outcomes were also recorded. Following overnight fasting (at least eight hours), 5 mL of venous blood was collected from each participant into ethylenediaminetetraacetic acid (EDTA) tubes. Plasma and erythrocyte fractions were separated by centrifugation at 3000 rpm for 10 minutes at 4 °C.

Oxidative Stress Biomarkers

CAT activity was determined spectrophotometrically based on the decomposition of hydrogen peroxide at 230 nm, whereas SOD activity was measured using the inhibition of nitroblue tetrazolium reduction at 505 nm [10,11]. MDA, an indicator of lipid peroxidation, was quantified using the thiobarbituric acid-reactive substances (TBARS) method [12].

Nitrosative Stress Biomarkers

NO levels were measured by the Griess reaction, while 3-NTx concentrations were determined spectrophotometrically as a marker of protein nitration [13,14].

All measurements were performed in duplicate in the Research Laboratory of the Department of Medical Biochemistry, Kahramanmaraş Sütçü İmam University Faculty of Medicine, using validated protocols.

CAT activity was measured using a commercially available spectrophotometric assay kit (Cayman Chemical, Ann Arbor, MI, USA), with an analytical sensitivity of 0.1 U/mL. The intra- and inter-assay coefficients of variation (CVs) were <5% and <8%, respectively. SOD activity was determined using a colorimetric assay kit based on the inhibition of nitroblue tetrazolium reduction (Cayman Chemical, USA), with a detection limit of 0.05 U/mL and intra- and inter-assay CVs of <6% and <9%, respectively. MDA levels were quantified using the thiobarbituric acid-reactive substances (TBARS) assay (Sigma-Aldrich, St. Louis, MO, USA), with a sensitivity of 0.1 µmol/L and intra- and inter-assay CVs of <7% and <10%, respectively. NO concentrations were measured using a colorimetric Griess reaction-based assay kit (Cayman Chemical, USA), with a detection limit of 1.0 µmol/L and CVs below 8%. Serum 3-nitrotyrosine (3-NTx) levels were measured using a commercially available enzyme-linked immunosorbent assay (ELISA) kit (Abcam, Cambridge, UK), with a minimum detectable concentration of 0.1 ng/mL. The intra- and inter-assay CVs were <6% and <9%, respectively. All assays were performed in duplicate according to the manufacturer’s protocols, and calibration curves were generated using certified standards provided with each kit.

Occupational exposure was defined as active employment in radiodiagnostic laboratories with routine contact with ionizing radiation sources. Work duration was recorded as a descriptive indicator of occupational history and potential cumulative exposure, rather than as a precise quantitative measure of individual radiation dose.

The study protocol was approved by the Non-interventional Clinical Research Ethics Committee of Kahramanmaraş Sütçü İmam University (Date: February 1, 2021, Decision No: 88). Written informed consent was obtained from all participants prior to enrollment, and the study was conducted in accordance with the Declaration of Helsinki.

Statistical analysis

Data were analyzed using IBM SPSS Statistics for Windows, version 25 (IBM Corp., Armonk, NY, USA). Continuous variables were expressed as mean ± standard deviation (SD) or median (interquartile range) as appropriate. Categorical variables were presented as frequencies and percentages. Intergroup comparisons were performed using the independent samples t-test or Mann-Whitney U test for continuous variables and chi-square test for categorical variables. Pearson’s correlation analysis was applied to evaluate the relationship between duration of occupational exposure and oxidative/nitrosative stress biomarkers. A p-value <0.05 was considered statistically significant.

## Results

A total of 200 participants were included in the study, comprising 100 radiodiagnostic laboratory personnel (exposed group) and 100 healthy individuals without occupational radiation exposure (control group). The mean age of the exposed group was 35.9 ± 7.1 years, while the control group had a mean age of 36.2 ± 6.8 years. The proportion of male participants was similar in both groups (70% vs. 68%). There were no statistically significant differences between the groups in terms of age or sex distribution (p=0.742 and p=0.815, respectively). The average duration of employment in radiodiagnostic laboratories among the exposed group was 10.9 ± 7.1 years, reflecting occupational history rather than measured cumulative radiation dose. Regarding radiation protection practices, 69% of the exposed group reported regular use of lead aprons, 59% reported using thyroid shields, and 80% reported using personal dosimeters (Table 1).

**Table 1:** Demographic characteristics of study participants

Variable	Exposed group (n=100)	Control group (n=100)	p-value
Age (years)	35.9 ± 7.1	36.2 ± 6.8	0.742
Sex (Male %)	70 (70%)	68 (68%)	0.815
Work duration (years)	10.9 ± 7.1	–	–
Lead apron use (%)	69 (69%)	–	–
Thyroid shield use (%)	59 (59%)	–	–
Dosimeter use (%)	80 (80%)	–	–

Oxidative and nitrosative stress biomarkers

Comparisons of oxidative and nitrosative stress biomarkers between groups are presented in Table 2. CAT activity was significantly lower in the exposed group compared to the controls (18.25 ± 4.07 vs. 22.46 ± 4.34 U/mg Hb, p=0.001). SOD activity was significantly higher in the exposed group (210.52 ± 32.38 vs. 180.21 ± 29.71 U/mg Hb, p=0.004). MDA levels, a marker of lipid peroxidation, were significantly elevated in exposed workers (4.84 ± 1.21 vs. 3.12 ± 0.92 µmol/L, p=0.002). NO concentrations were significantly higher in the exposed group compared to controls (35.74 ± 6.81 vs. 26.05 ± 5.67 µmol/L, p=0.003). Levels of 3-NTx were also significantly elevated in the exposed group (18.41 ± 3.59 vs. 12.89 ± 3.14 ng/mL, p=0.001). See Appendix E.

**Table 2:** Oxidative and nitrosative stress biomarkers in exposed and control groups CAT: catalase; SOD: superoxide dismutase; MDA: malondialdehyde; NO: nitric oxide; 3-NTx: 3-nitrotyrosine

Biomarker	Exposed group (n=100)	Control group (n=100)	t-value	p-value
CAT (U/mg Hb)	18.25 ± 4.07	22.46 ± 4.34	5.12	0.001
SOD (U/mg Hb)	210.52 ± 32.38	180.21 ± 29.71	6.45	0.004
MDA (µmol/L)	4.84 ± 1.21	3.12 ± 0.92	5.98	0.002
NO (µmol/L)	35.74 ± 6.81	26.05 ± 5.67	6.01	0.003
3-NTx (ng/mL)	18.41 ± 3.59	12.89 ± 3.14	6.75	0.001

**Figure 1:**
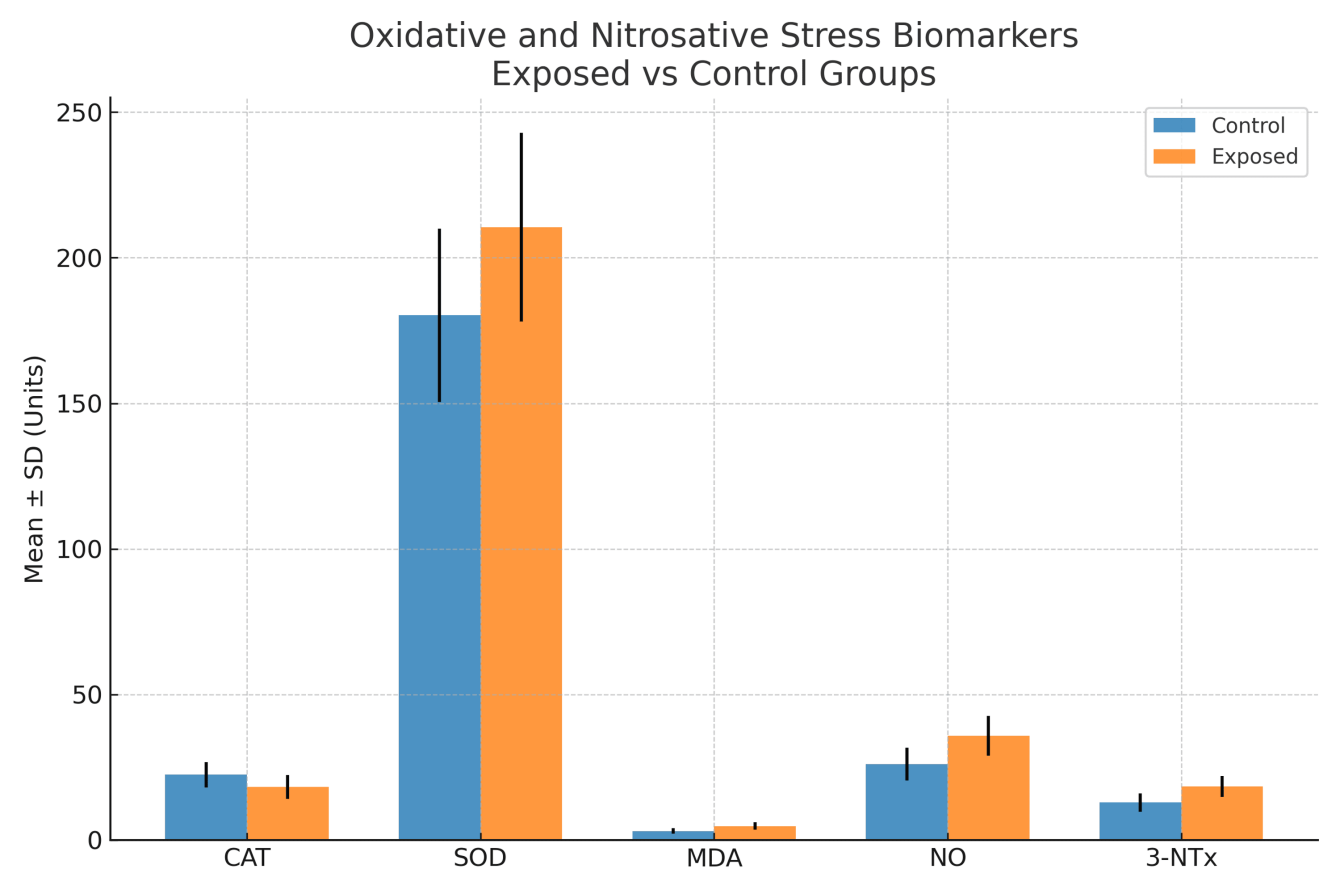
Comparison of oxidative and nitrosative stress biomarkers between exposed and control groups Bar graph showing mean ± SD values of oxidative and nitrosative stress biomarkers in radiodiagnostic laboratory personnel (exposed) and healthy controls. Catalase (CAT) activity was significantly lower in the exposed group, whereas superoxide dismutase (SOD) activity, malondialdehyde (MDA), nitric oxide (NO), and 3-nitrotyrosine (3-NTx) levels were significantly higher compared to controls. Error bars represent standard deviation.

These findings indicate that occupational exposure to ionizing radiation was associated with increased oxidative and nitrosative stress, reflected by elevated MDA, NO, and 3-NTx levels, increased SOD activity, and decreased CAT activity (Figure 1).

## Discussion

In this study, we investigated oxidative and nitrosative stress biomarkers in radiodiagnostic laboratory personnel exposed to ionizing radiation (IR) in the Eastern Mediterranean region of Türkiye. Our findings demonstrate that exposure to IR is associated with decreased CAT activity, increased SOD activity, and elevated levels of MDA, NO, and 3-NTx. These results suggest that occupational IR exposure disrupts the oxidant-antioxidant balance, leading to oxidative and nitrosative stress. The observed reduction in CAT activity in exposed workers may reflect enzyme consumption as a consequence of increased hydrogen peroxide formation during radiation-induced oxidative stress. Similar decreases in CAT activity have been reported among medical radiation workers, supporting the hypothesis that chronic low-dose IR may impair antioxidant defenses [15]. Conversely, we observed an increase in SOD activity, which may represent a compensatory upregulation in response to excess superoxide anion production. Previous studies have also shown that SOD activity is often elevated in individuals with chronic oxidative stress as a cellular adaptation mechanism [16].

MDA levels were significantly higher in the exposed group, indicating enhanced lipid peroxidation. This finding aligns with earlier research demonstrating that IR exposure increases lipid peroxidation products, which serve as reliable markers of oxidative damage [17,18]. Lipid peroxidation can compromise cell membrane integrity, promote mutagenesis, and ultimately contribute to carcinogenesis, underscoring its clinical relevance in occupational radiation safety. We also found significant elevations in NO and 3-NTx levels in radiation-exposed workers. NO is a reactive nitrogen species with dual physiological and pathological roles. While NO at physiological concentrations regulates vasodilation and neurotransmission, its overproduction leads to peroxynitrite formation, which in turn nitrates tyrosine residues to form 3-NTx. Elevated 3-NTx levels observed in our study indicate protein nitration and nitrosative stress. Prior investigations have reported similar increases in nitrotyrosine levels among individuals exposed to IR, further supporting our findings [19,20].

Taken together, our results suggest that chronic occupational IR exposure induces a state of persistent oxidative and nitrosative stress. This imbalance may have long-term health implications. Oxidative stress has been implicated in the pathogenesis of cancer, cardiovascular disease, and neurodegenerative disorders, conditions that may occur with higher prevalence among healthcare workers exposed to radiation [21]. Nitrosative stress, particularly peroxynitrite-mediated protein modifications, has been associated with autoimmune phenomena and tissue injury, raising concerns about possible systemic effects in chronically exposed populations [22]. Our findings are consistent with several international reports. Ionizing radiation is known to trigger cellular damage through mechanisms such as oxidative stress and radiation-induced bystander effects [23]. Mitochondria function as key regulators of cellular stress responses and apoptosis, underscoring their critical role in pathways of cellular injury [24]. Collectively, these studies strengthen the evidence that even low-dose occupational radiation exposure, when accumulated over years, may have measurable biological effects.

Another important implication of our study is the observed gap in radiation safety practices. Despite high reported rates of dosimeter and protective equipment usage, biomarker alterations were still evident. This highlights the need for regular training programs, stricter monitoring of cumulative doses, and potential dietary or pharmacological interventions aimed at reinforcing antioxidant defenses. Antioxidant supplementation, such as vitamins C and E, has been suggested as a potential protective strategy against radiation-induced oxidative stress, although more randomized controlled trials are needed to validate their efficacy [25]. Nevertheless, this study has certain limitations. First, its cross-sectional design precludes establishing causality between radiation exposure and oxidative/nitrosative stress. Second, we did not assess genetic polymorphisms of antioxidant enzymes, which may influence inter-individual variability in response to IR. Third, the study population was limited to three provinces, which may not fully represent all radiology workers in Türkiye. Future longitudinal studies with larger cohorts and mechanistic investigations are warranted to confirm these findings.

Another important consideration is the external validity of the present findings. Radiation safety practices, including the routine use of personal protective equipment, availability of dosimeters, institutional safety culture, and adherence to radiation protection guidelines, may vary substantially between healthcare institutions and countries. In addition, differences in equipment age, maintenance standards, and procedural workload (particularly in high-volume interventional radiology units) may influence cumulative occupational exposure levels. As the present study was conducted in selected centers within the Eastern Mediterranean region of Türkiye, these regional and institutional characteristics should be taken into account when extrapolating the results to other settings. Therefore, caution is warranted when generalizing our findings to radiodiagnostic personnel working under different safety protocols or technological conditions.

Importantly, the present study did not evaluate DNA damage, clinical outcomes, or symptom-biomarker associations. Therefore, the findings should not be interpreted as evidence of increased disease risk but rather as biochemical alterations associated with occupational exposure to radiation-related work environments.

An additional limitation of this study is the heterogeneity of the radiation-exposed group. Radiodiagnostic personnel encompass diverse professional roles, including interventional radiology staff, computed tomography technicians, and conventional X-ray operators, who are known to differ substantially in terms of radiation dose intensity and exposure patterns. Due to the limited sample size within each occupational category and the lack of comprehensive individual cumulative dose data, subgroup analyses according to professional role were not feasible in the present study. This heterogeneity may have contributed to variability in biomarker levels and should be considered when interpreting the results. Future studies with larger sample sizes, standardized dosimetry records, and stratified analyses are needed to elucidate role-specific oxidative and nitrosative stress responses.

Several limitations of this study should be acknowledged. First, the cross-sectional design limits the ability to establish temporal or causal relationships between occupational ionizing radiation exposure and alterations in oxidative and nitrosative stress biomarkers. Consequently, it cannot be determined whether the observed biomarker elevations preceded radiation exposure or developed as a result of cumulative exposure over time. In addition, oxidative and nitrosative stress biomarkers are known to exhibit biological variability and may fluctuate over time. Longitudinal studies incorporating repeated biomarker measurements and cumulative dose assessment are, therefore, warranted to provide more definitive evidence regarding temporal dynamics and causality.

Another important limitation relates to exposure assessment. In this study, occupational exposure was categorized based on employment in radiodiagnostic laboratories, and work duration was used as a surrogate indicator of occupational history rather than a direct measure of radiation dose. Therefore, the observed biomarker alterations cannot be attributed exclusively to ionizing radiation exposure, as other occupational or environmental factors (such as workload, shift patterns, psychosocial stress, or non-radiation-related chemical exposures) may also contribute. Consequently, the findings should be interpreted as associations with radiation-related occupational environments rather than definitive radiation-induced effects.

In summary, our study provides evidence that occupational exposure to ionizing radiation is associated with increased oxidative and nitrosative stress, as reflected by altered biomarker profiles. These findings underscore the importance of reinforcing radiation protection strategies and considering antioxidant interventions to safeguard the health of healthcare workers.

## Conclusions

This study showed that radiodiagnostic laboratory personnel chronically exposed to ionizing radiation had significantly altered oxidative and nitrosative stress biomarker profiles. Lower catalase activity, higher superoxide dismutase activity, and elevated levels of malondialdehyde, nitric oxide, and 3-nitrotyrosine suggest a disrupted oxidant-antioxidant balance.

These findings highlight the potential long-term health risks of occupational radiation exposure and emphasize the importance of strict radiation protection measures. Routine biomarker monitoring and antioxidant-based preventive strategies may help mitigate adverse effects, but further longitudinal studies are needed to confirm these observations.

## Appendices

Appendix A

**Table 3:** Kahramanmaraş group HCAT: homocysteine; HSOD: superoxide dismutase; HMDA: malondialdehyde, HNO: nitric oxide; HNTX: nitrotyrosine

Statistic	HCAT	HSOD	HMDA	HNO	HNTX
N (valid)	25	25	25	25	25
N (missing)	75	75	75	75	75
Mean	5.2204	3942.68	6.7848	76.3062	136.66
Std. error of mean	0.38761	208.2065	0.32742	1.63252	2.26437
Median	5.02	3984.0	6.77	76.88	136.25
Std. deviation	1.93806	1041.03249	1.63708	8.16261	11.32184
Minimum	1.77	2037.0	3.54	59.0	100.43
Maximum	8.99	5877.0	10.03	91.03	155.04

Appendix B

**Table 4:** Adana group HCAT: homocysteine; HSOD: superoxide dismutase; HMDA: malondialdehyde, HNO: nitric oxide; HNTX: nitrotyrosine

Statistic	HCAT	HSOD	HMDA	HNO	HNTX
N (valid)	50	50	50	50	50
N (missing)	50	50	50	50	50
Mean	4.8114	3980.88	7.714	75.7606	133.825
Std. error of mean	0.26341	126.14811	0.24639	1.14968	1.29631
Median	4.24	3920.0	8.42	73.365	133.79
Std. deviation	1.8626	892.00182	1.74224	8.12948	9.16629
Minimum	2.65	2077.0	3.77	60.0	110.95
Maximum	10.02	6200.0	9.88	92.45	151.06

Appendix C

**Table 5:** Hatay group HCAT: homocysteine; HSOD: superoxide dismutase; HMDA: malondialdehyde, HNO: nitric oxide; HNTX: nitrotyrosine

Statistic	HCAT	HSOD	HMDA	HNO	HNTX
N (valid)	25	25	25	25	25
N (missing)	75	75	75	75	75
Mean	5.2284	3817.92	5.4028	74.5996	134.2568
Std. error of mean	0.45153	134.54646	0.2604	0.71114	1.63881
Median	4.65	3625.0	5.18	73.27	134.71
Std. deviation	2.25764	672.7323	1.30202	3.5557	8.19407
Minimum	2.34	3000.0	3.84	69.55	120.59
Maximum	10.98	5300.0	8.95	81.03	147.0

Appendix D

**Table 6:** Questionnaire used for data collection

Questionnaire used for data collection
1. Age
2. Sex
3. Height
4. Weight
5. Marital Status
6. Education Level
7. Household size
8. İncome
8. Smoking
9. Alcohol consumption habits
10. Chronic diseases
11. Occupational history
12. Radiation protection measures
13. Reproductive health
14. Common complaints
-Fatigue
-Headache
-Irritability
-insomnia

Appendix E

**Table 7:** Healthy controls HCAT: homocysteine; HSOD: superoxide dismutase; HMDA: malondialdehyde, HNO: nitric oxide; HNTX: nitrotyrosine

Statistic	CAT	SOD	MDA	NO	NTX
N (valid)	50	50	50	50	50
N (missing)	50	50	50	50	50
Mean	11.749	2418.26	2.1794	59.5286	67.2426
Std. error of mean	0.31981	103.38287	0.06925	0.3744	1.70767
Median	12.035	2560.5	2.155	60.025	67.42
Std. deviation	2.26141	731.02731	0.48967	2.64742	12.07504
Minimum	6.55	1036.0	1.22	53.88	40.82
Maximum	15.05	3865.0	3.02	64.49	88.02
